# ID 115 - Cost-Effectiveness and Budget Impact Analysis of Expanding Mammography Screening in Brazil

**DOI:** 10.5327/2237-9622.2025.v34s1.115

**Published:** 2025-11-25

**Authors:** Daniela Gorski, Caroline Mensor Folchini, Layssa Andrade Oliveira, Vinicius Lins Ferreira, Astrid Wiens, Fernanda Stumpf Tonin, Roberto Pontarolo

**Keywords:** cost-effectiveness, budget impact analysis, breast cancer, screening procedures

## Abstract

**Introdução:**

Mammography screening for breast cancer became public policy in Brazil in 2004, with current guidelines recommending biennial mammograms for asymptomatic women aged 50-691. The Brazilian Society of Mastology recently recommends for annual mammograms starting at age 40, claiming a 13-25% reduction in breast cancer mortality2. This study aims to assess the cost-effectiveness and budget impact of expanding screening to women aged 40-49 and those over 69 at the Brazilian Unified Health System (SUS).

**Método:**

A decision tree coupled with a Markov model was developed to compare the expansion of screening to asymptomatic women aged 40-49 and those over 69 compared to usual care without screening for this age. In the screening group, after mammography the result could indicate cancer or not. Health states in Markov model for women developing cancer included remission, local recurrence, distant recurrence and death. A lifetime horizon with annual cycles was considered, applying a 5% discount rate to both costs and outcomes. Clinical outcomes measured were quality-adjusted life years (QALYs) and life years (LY). The analysis adopted the SUS perspective, considering direct costs of mammography [Brazilian public healthcare system table of procedures and medications (SIGTAP)] and breast cancer treatments (surgery, radiotherapy, chemotherapy). The budgetary impact was assessed using demand projections and population data from the Brazilian National Institute of Geography and Statistics (IBGE) and the National Cancer Institute's Annual Report.

**Resultados:**

The cost-effectiveness analysis revealed that the expanded screening strategy was cost-effective compared to usual care. For women aged 40-49, the incremental cost-effectiveness ratio (ICER) was R$57,000 per QALY and R$37,000 per LY. For women over 69, the ICER was R$44,000 per QALY and R$28,000 per LY. In the budget impact analysis, it was estimated that around 16 million women would undergo this procedure through the SUS (considering market share 100%). The results showed that expanding screening would significantly increase costs, reaching incremental costs of R$96 million by the first year and totaling R$512 million over the five-year period.

**Conclusão:**

The findings suggest that expanding mammography screening to women aged 40-49 and over 69 could be cost-effective, with ICER values below the acceptable threshold for serious diseases3. Moreover, implementing this expanded program would require carefully addressing operational challenges, including additional equipment, maintenance, and staffing across the country. In addition to women's greater exposure to ionizing radiation. The literature has shown that exposure between the ages of 40 and 74 increases the risk of breast cancer by 0.11% and 0.29% for women with and without a family history of cancer4. The results highlight the need to balance clinical benefits with financial implications, emphasizing ongoing evaluation of evidence, cost monitoring, and the use of further advanced techniques such as value-of-information analysis, as seen in other countries exploring similar expansions.

